# The *Aux/IAA* gene *rum1* involved in seminal and lateral root formation controls vascular patterning in maize (*Zea mays* L.) primary roots

**DOI:** 10.1093/jxb/eru249

**Published:** 2014-06-13

**Authors:** Yanxiang Zhang, Anja Paschold, Caroline Marcon, Sanzhen Liu, Huanhuan Tai, Josefine Nestler, Cheng-Ting Yeh, Nina Opitz, Christa Lanz, Patrick S. Schnable, Frank Hochholdinger

**Affiliations:** ^1^INRES, Institute of Crop Science and Resource Conservation, Crop Functional Genomics, University of Bonn, Friedrich-Ebert-Allee 144, 53113 Bonn, Germany; ^2^Department of Agronomy, Iowa State University, Ames 50011-3650, Iowa, USA; ^3^Center for Plant Genomics, Iowa State University, Ames 50011-3650, Iowa, USA; ^4^Department of Molecular Biology, Max-Planck-Institute for Developmental Biology, 72076 Tuebingen, Germany

**Keywords:** Auxin, lateral roots, lignification, RNA-Seq, RUM1, vasculature, xylem.

## Abstract

The maize (*Zea mays* L.) Aux/IAA protein RUM1 (ROOTLESS WITH UNDETECTABLE MERISTEMS 1) controls seminal and lateral root initiation. To identify RUM1-dependent gene expression patterns, RNA-Seq of the differentiation zone of primary roots of *rum1* mutants and the wild type was performed in four biological replicates. In total, 2 801 high-confidence maize genes displayed differential gene expression with Fc ≥2 and FDR ≤1%. The auxin signalling-related genes *rum1*, *like-auxin1* (*lax1*), *lax2*, (*nam ataf cuc 1* *nac1*), the *plethora* genes *plt1* (*plethora 1*), *bbm1* (*baby boom 1*), and *hscf1* (*heat shock complementing factor 1*) and the *auxin response factor*s *arf8* and *arf37* were down-regulated in the mutant *rum1*. All of these genes except *nac1* were auxin-inducible. The maize *arf8* and *arf37* genes are orthologues of *Arabidopsis MP/ARF5* (*MONOPTEROS*/*ARF5*), which controls the differentiation of vascular cells. Histological analyses of mutant *rum1* roots revealed defects in xylem organization and the differentiation of pith cells around the xylem. Moreover, histochemical staining of enlarged pith cells surrounding late metaxylem elements demonstrated that their thickened cell walls displayed excessive lignin deposition. In line with this phenotype, *rum1*-dependent mis-expression of several lignin biosynthesis genes was observed. In summary, RNA-Seq of RUM1-dependent gene expression in maize primary roots, in combination with histological and histochemical analyses, revealed the specific regulation of auxin signal transduction components by RUM1 and novel functions of RUM1 in vascular development.

## Summary

RNA-Seq of RUM1-dependent gene expression in maize primary roots, in combination with histological analyses, highlighted the regulation of auxin signal transduction by RUM1 and its role in vascular development.

## Introduction

Maize (*Zea mays* L.) plays an important agronomic role as feed, food, and source of bioethanol. The complex root system of maize facilitates water and nutrient uptake and anchorage of the plant (Aiken and Smucker, 1996). The embryonic root system of maize consists of a primary root and a variable number of seminal roots. The post-embryonic root system comprises lateral and shoot-borne roots. In all maize root types lateral roots are initiated from phloem pole pericycle and endodermis cells (Hochholdinger *et al.*, 2004a).

The plant hormone auxin is a key regulator of many aspects of plant development. In roots, auxin controls lateral root development (Jansen *et al.*, 2012; Peret *et al*., 2009a, b), root architecture (Overvoorde *et al.*, 2010) and vascular development (Wilson *et al.*, 1991; Uggla *et al.*, 1996; Mattsson *et al.*, 1999). It was suggested that polar auxin transport controls lateral root initiation in maize (Jansen *et al.*, 2012). Moreover, an auxin maximum was observed from late metaxylem to protoxylem in cells surrounding protophloem cells in the transgenic maize marker line DR5-RFP (Jansen *et al.*, 2012). This auxin maximum is likely to be essential for lateral root positioning and initiation. By contrast, inhibition of polar transport prevents an auxin response maximum from xylem to phloem pole cells in an anticlinal orientation and results in disorganized vascular tissues (Mattsson *et al.*, 1999; Jansen *et al.*, 2012).

Central regulators of auxin signalling include the *T*ransport *I*nhibitor *R*esponse *1* (TIR1) protein, members of the TIR1-like *A*uxin *F*-*B*ox (AFBs) family, *Aux*in/*I*ndole *A*cetic *A*cid (Aux/IAA) proteins, and *A*uxin *R*esponse *F*actor (ARF) proteins (Mockaitis and Estelle, 2008). At low intracellular auxin concentrations, Aux/IAA proteins act as transcriptional repressors that interact with ARF proteins via their domains III and IV. The ARF proteins of these complexes interact with *Aux*in *R*esponsive *E*lements (AuxREs) in the promoters of downstream genes, thereby repressing their transcription. By contrast, high auxin levels stabilize interactions between Aux/IAA proteins and SCF^TIR1^ E3 ubiquitin–ligase complexes, resulting in the degradation of Aux/IAA proteins by the 26S proteasome (Gray *et al.*, 2001; Tian *et al.*, 2002; Woodward and Bartel, 2005; Tan *et al.*, 2007). As a consequence, ARF proteins released from the Aux/IAA interactions can promote auxin-responsive target gene transcription.

The semi-dominant maize *rum1* (*rootless with undetectable meristem 1*) mutant is blocked in the initiation of embryonic seminal roots and post-embryonic lateral roots of the primary root (Woll *et al.*, 2005). The mutant *rum1* displays several auxin-related defects in root development. While exogenous auxin (αNAA) application induced additional lateral roots in wild-type primary roots, αNAA did not initiate any lateral roots in *rum1* (Woll *et al.*, 2005). Moreover, polar auxin transport in *rum1* primary roots was reduced by 83% compared with wild-type primary roots while it was not affected in the coleoptile of the mutant *rum1* (Woll *et al.*, 2005). The *rum1* gene (Genbank AC: GRMZM2G037368) encodes ZmIAA10, a member of the Aux/IAA protein family (Wang *et al.*, 2010). RUM1 interacts with the transcriptional activators ARF25 and ARF34 (von Behrens *et al.*, 2011). The mutated rum1 protein lacks 24 amino acids including the degron motif ‘GWPPV’ in domain II of rum1. Therefore, rum1 cannot interact with the SCF^TIR1^ E3 ubiquitin–ligase complexes which prevents its ubiquitin-mediated proteasomal degradation and resulting in constitutive repression of downstream gene expression (von Behrens *et al.*, 2011). In the present study, genes differentially expressed between wild-type and *rum1* primary roots were identified via RNA-Seq suggesting direct or indirect regulation of these genes by RUM1. In combination with histological and histochemical analyses, a RUM1-dependent gene network was identified and novel functions of RUM1 in vascular development were revealed.

## Materials and methods

### Plant material and growth conditions

Seeds of the F_8_-generation of the maize mutant *rum1* and its homozygous wild type obtained by seven cycles of selfing of heterozygous plants were used in these experiments. Seeds were sterilized with 6% hypochlorite under vacuum at 500 mPa for 5min, rinsed five times in distilled water, and germinated in paper rolls in a plant growth chamber at 28 °C with a 16/8h light/dark regime at 21 °C (Woll *et al.*, 2005). For the RNA-Seq experiment, primary roots, 2cm in length, of the mutant *rum1* and its homozygous wild type were harvested. Subsequently, 5mm of the root tip, including the meristematic and elongation zones, were removed. Hence, only the differentiation zone of these roots was analysed by RNA-Seq. For auxin induction experiments, 5-d-old seedlings of the maize inbred line B73 that were grown under the same conditions were treated with the auxin analogue αNAA (α-naphthyl acetic acid; working solution 5 µM) for 3h. The differentiation zone of the ~5cm primary roots was harvested after 0, 1, 2, and 3h of αNAA exposure (Taramino *et al.*, 2007) for subsequent analyses. For histological experiments, wild-type and *rum1* seedlings were grown in αNAA (0.1 µM) or the auxin transport inhibitor NPA (1-*N*-naphthylphthalamic acid, 10 µM).

### RNA extraction and RNA sequencing

Total RNA was extracted via the RNeasy Plant Mini Kit (Qiagen, Hilden, Germany) and 3–4 roots were pooled per replicate, subsequently treated with RNase-free DNaseI (Fermentas, St Leon-Roth, Germany). RNA quality of all samples was assayed via a Bioanalyzer (Agilent Technologies, Boeblingen, Germany). As suggested by Agilent only samples with an RIN (RNA integrity number ≥7 were used for downstream analyses. Per genotype (wild type versus *rum1*) or treatment (auxin induction) four biological replicates were surveyed. The cDNA libraries for RNA-Seq were constructed using the TruSeq RNA sample preparation kit (Illumina Inc., San Diego, CA, USA). Each of the four replicates of each genotype was bar coded with one of the Illumina indices AR001, AR003, AR008, and AR009. Subsequently, the four wild-type replicates were pooled and loaded onto lane three of a flow cell, while the four *rum1* replicates were pooled on lane four of a flow cell. These lanes were then sequenced using a Genome Analyzer II (Illumina Inc., San Diego, CA, USA) according to the manufacturer’s instructions resulting in 146bp single-end reads.

### RNA-Seq mapping and statistical analysis

RNA-Seq reads were trimmed according to Liu *et al.* (2012) and subsequently mapped to the B73 reference genome (ZmB73_RefGen_v2) (Schnable *et al.*, 2009) using GSNAP (Wu and Nacu, 2010). For subsequent analyses, only reads mapping uniquely (≤2 mismatches every 36bp and fewer than five bases for every 75bp as tails) to the reference genome were used. Genes with an average of at least five uniquely mapped reads across libraries and at least two samples with positive read counts (*n*=22 833) were tested for differential expression using the R package *QuasiSeq* (http://cran.r-project.org/web/packages/QuasiSeq). The 0.75 quartile of reads from each library was used as the normalization factor (Bullard *et al.*, 2010). The negative binomial *QLSpline* method implemented in the *QuasiSeq* package was used to compute a *p*-value for each gene (Lund *et al.*, 2012). A multiple test controlling approach (Nettleton *et al.*, 2006) was used to convert the *p*-values to *q*-values (Storey, 2002). Genes with FDR ≤1% and Fc ≥2 were declared to be differentially expressed (Li *et al.*, 2011). Subsequently, genes were classified into functional categories via MapMan (Thimm *et al.*, 2004). To determine if specific functional groups are overrepresented among the differentially expressed genes with reference to all expressed genes, the expected number of genes for each functional category was calculated based on the distribution of functional categories among all expressed genes. To determine if significantly more or less genes than expected were detected for each individual category a χ^2^ test for independence with Yates’ continuity correction was performed.

### qRT-PCR expression analyses

cDNA for qRT-PCR was synthesized from 500ng total RNA using the qScript cDNA SuperMix (Quanta Biosciences, Gaithersburg, MD, USA). qPCR was performed in a CFX384 Real-Time PCR detection system (Bio-Rad, Munich, Germany) for each of the four biological replicates in three technical replications in a total reaction volume of 8 µl using the MESA Green qPCR Mastermix Plus for SYBR Assay no ROX kit (Eurogentech, Cologne, Germany). Primers with an efficiency between 0.9 and 1.1 were used for qPCR, which was tested in a dilution series (1, 1/2, 1/4, 1/8, 1/16, 1/32, 1/64, 1/128). Gene expression for each genotype and each time point of auxin induction was assayed relative to *myosin* (Genbank AC: 486090G09.x1; primers: 486090G09.x1-5′; 486090G09.x1-3′) which has been previously used as a qPCR standard for expression analyses in maize roots (von Behrens *et al.*, 2011). The oligonucleotide primers *rum1*-fw and *rum1*-rv (*rum1*, GRMZM2G037368), *arf8*-fw and *arf8*-rv (*arf8*, GRMZM2G034840), *arf37*-fw and *arf37*-rv (*arf37*, GRMZM2G086949), *nac1*-fw and *nac1*-rv (*nac1*, GRMZM2 G081930), *lax1*-fw and *lax1*-rv (*lax1*, GRMZM2G129413), *lax2*-fw and *lax2*-rv (*lax2*, GRMZM2G149481), *plt1*-fw and *plt1*-rv (*plt1*, GRMZM2G141638), *bbm1*-fw and *bbm1*-rv (*bbm1*, GRMZM2 G366434), *hscf1*-fw and *hscf1*-rv (*hscf1*, GRMZM2G139082), *cad*-fw and *cad*-rv (*cad*, GRMZM2G443445), and *f5h*-fw and *f5h*-rv (*f5h*, GRMZM2G100158) were used for surveying expression of these genes (see Supplementary Table S5 at *JXB* online). Differential gene expression was determined by Student′s *t* tests (*p ≤0.05; **p ≤0.01; ***p ≤0.001; *n*=4).

### Histology and histochemistry

Feulgen staining of whole roots was performed as previously described by Woll *et al.* (2005). For histological analyses and subsequent lignin staining, root fragments of the differentiation zone of the primary root were fixed in 4% paraformaldehyde in 1× phosphate buffer (containing 0.01M Na_2_HPO_4_ and 0.01M NaH_2_PO_4_, pH 7) for 2h under vacuum at 100 mPa. Subsequently, the root fragments were sectioned by hand, mounted with water and then transferred into 65% glycerol. Transverse sections were analysed under bright field using a dissection microscope (PixCell IIe System, Zeiss). For lignin staining, transverse sections were incubated in 1% phloroglucinol in 12% HCl for 10min, then transferred into acid solution (75% glycerol in 10% H_2_SO_4_) and observed under bright field conditions using a dissection microscope (PixCell IIe System, Zeiss).

## Results

### Comparative RNA-Seq analysis of *rum1* and wild-type primary roots

**Fig. 1.**
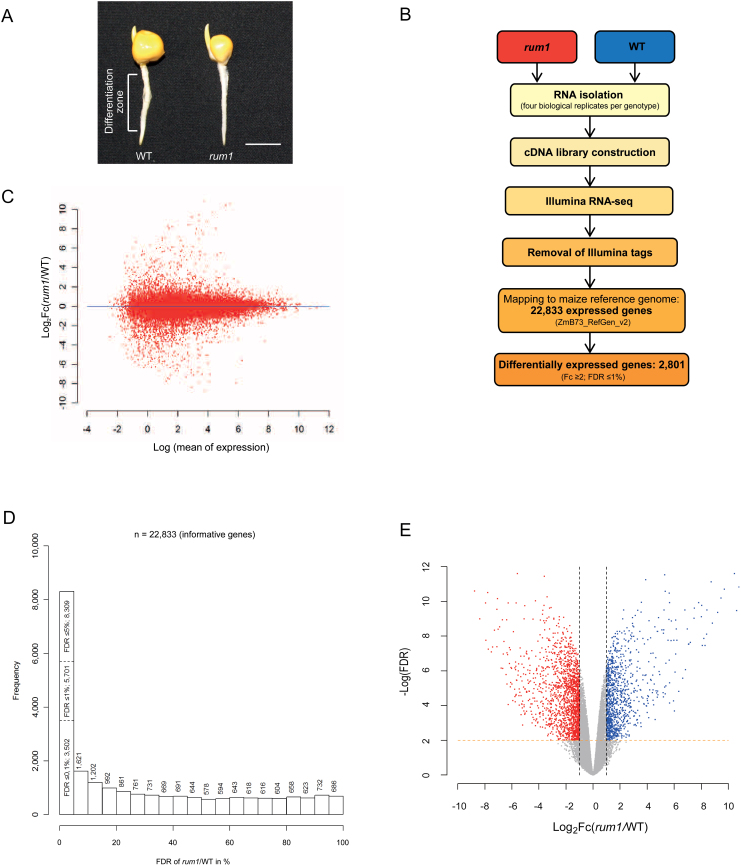
(A) Phenotype of 2cm primary roots of homozygous wild type (left) and *rum1* (right). The differentiation zone of the primary roots was subjected to RNA-Seq experiments. Lateral root primordia do not develop at this stage (Woll *et al.*, 2005). Scale bar: 1cm. (B) Flow chart of the Illumina RNA-Seq experimental design. (C) MA-similar plot providing an overview of the correlation of gene expression changes versus expression intensity by plotting the log_2_ of the fold change of *rum1*/wild type against the log_2_ of the mean of gene expression in *rum1* and the wild type from RNA-Seq data. Each dot represents an expressed gene. (D) Histogram of FDR-values resulting from the comparison of gene expression between the *rum1* mutant and wild-type primary roots. (E) Volcano plot displaying differential gene expression patterns between *rum1* and the wild type. The *Y*-axis denotes negative log_10_ FDR of each expressed gene. The *X*-axis denotes log_2_-fold changes of *rum1*/wild type. Each dot represents an expressed gene. Red dots denote down-regulated genes in *rum1*, blue dots denote up-regulated genes in *rum1* with cut-off thresholds of Fc ≥2; FDR ≤1%. (This figure is available in colour at *JXB* online.)

The maize *rum1* mutant is impaired in lateral root initiation in the primary root. To understand the molecular network regulated by the Aux/IAA protein RUM1, ~2cm long primary roots of the mutant *rum1* and homozygous wild-type siblings were subjected to an RNA-Seq analysis (Fig. 1A). At this early developmental stage no lateral root primordia were detectable in primary roots of wild-type seedlings by Feulgen staining (data not shown). Hence, at this stage, primary roots of wild-type and *rum1* were morphologically indistinguishable. Lateral roots in maize are formed in the differentiation zone of roots. Therefore, only the differentiation zone of primary roots, i.e. primary roots, after the removal of 5mm of the root tip which comprises the meristematic zone and most of the elongation zone, were used for subsequent transcriptome profiling. A flow chart of the RNA-Seq experiment is provided in Fig. 1B. Sequencing of four biological replicates of wild-type and *rum1* libraries yielded, on average, ~15 million 146bp single-end reads per sample (see Supplementary Table S1 at *JXB* online). After removal of bar-code tags from *rum1* and wild-type reads, on average, 76% and 73% of the trimmed reads mapped to unique positions of the maize B73 reference genome (ZmB73_RefGen_v2; see Supplementary Table S1 at *JXB* online), respectively. Of these reads, 94–95% were associated with high-confidence gene models of the ‘filtered gene set’ (4a.53_v2; maizesequence.org).

### Identification of differentially expressed genes

The RNA-Seq reads from the differentiation zone of 2-cm-long maize primary roots aligned to 22 833 of 39 656 (58%) genes in the high confidence ‘filtered gene set’ (see Supplementary Table S2 at *JXB* online). The distribution of fold-changes versus the mean of gene expression in the differentiation zone of primary roots between *rum1* and the wild type demonstrated that differential gene expression was observed for genes with both low and high expression levels (Fig. 1C). In total, 8 309 (FDR ≤5%), 5 701 (FDR ≤1%), and 3 502 (FDR ≤0.1%) genes displayed differential expression at different significance levels irrespective of their fold-changes (Fig. 1D). Hence, these classes represented 36%, 25%, and 15% of all genes for which expression was detected. At FDR ≤1%, 2 801 (12% of all) genes displayed a Fc (fold change) ≥2 (see Supplementary Table S2 at *JXB* online), including 1 741 genes that were down-regulated (Fig. 1E, red dots) and 1 060 genes that were up-regulated in the *rum1* mutant (Fig. 1E, blue dots). Of those genes, 1 723 (62%) were functionally annotated using the MapMan software (see Supplementary Table S3 at *JXB* online), including 205 genes that were assigned to more than one functional class. To identify over- and underrepresented functional classes among the differentially expressed genes, the expected number of genes for each functional class was calculated based on all expressed genes and subsequently compared with the detected number of genes in this class. This analysis revealed that significantly more genes than expected (*p* ≤0.001) were assigned to the classes *photosynthesis*, *cell wall*, *metal handling*, *secondary metabolism, miscellaneous*, and *DNA* (see Supplementary Table S4 at *JXB* online).

### qRT-PCR confirmation of differentially expressed genes

**Fig. 2.**
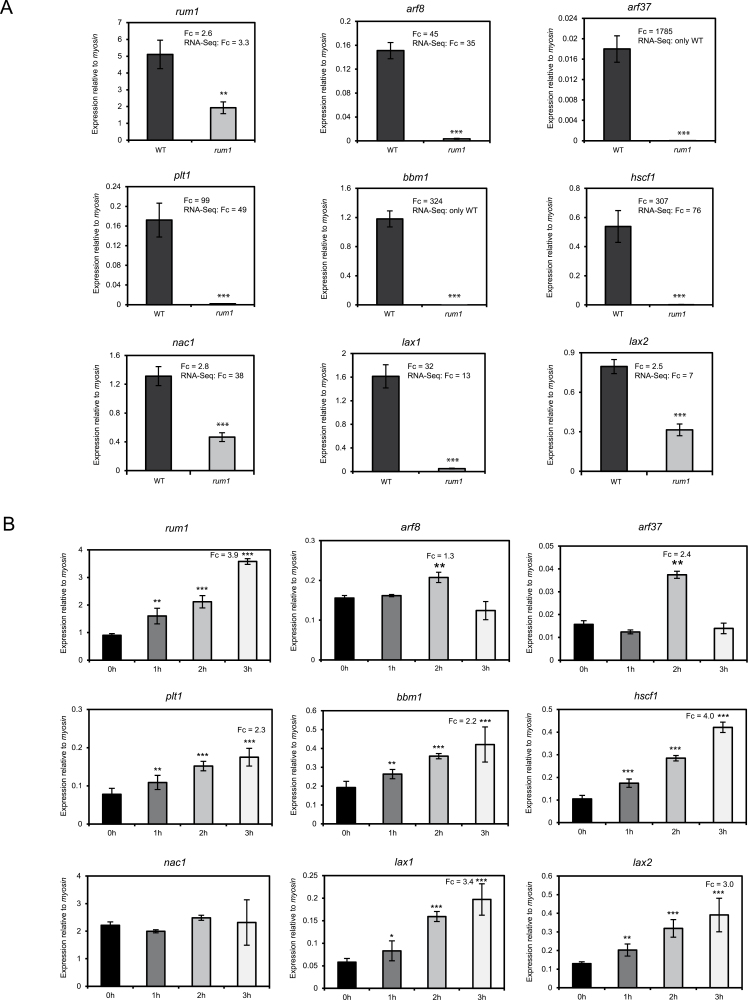
(A) Confirmation of differential gene expression of nine genes involved in auxin signal transduction using qRT-PCR. Each of these genes was differentially expressed between *rum1* and the wild type in the RNA-Seq experiment. In each graph fold-changes (Fc; *rum1*/wild type) from the qRT-PCR and the RNA-Seq experiment are displayed. (B) Auxin inducibility was tested for the nine auxin-related genes by a qRT-PCR time-course experiment with reference to time point 0 h. Wild-type primary roots (5-d-old) were treated with αNAA and harvested at 0, 1, 2, and 3h of exposure. Statistical analyses were performed with a two-sided Student’s *t* test (*p ≤0.05; **p ≤0.01; ***p ≤0.001).

To confirm the RNA-Seq results independently, expression levels of a subset of differentially expressed genes related to auxin signalling were analysed by qRT-PCR. Consistent with prior results (von Behrens *et al.*, 2011), the *Aux/IAA* gene *rum1* (GRMZM2G037368), used as a positive control, was down-regulated in the *rum1* mutant compared with the wild type (Fig. 2A). Moreover, three *plethora* (*plt*) genes *plt1* (GRMZM2G141638), *bbm1* (GRMZM2G366434), and *hscf1* (GRMZM2G139082) were down-regulated in *rum1* (Fig. 2A). Furthermore, two auxin response factors *arf8* (GRMZM2G034840) and *arf37* (GRMZM2G086949), two auxin influx transporters *like*-*aux1* (*lax1*, GRMZM2G129413) and *lax2* (GRMZM2G149481), and a *nac1* (GRMZM2G081930) gene were down-regulated in *rum1* compared with wild-type primary roots.

### Auxin inducibility of differentially expressed genes involved in auxin signal transduction

To test the auxin inducibility of these auxin signal transduction genes, time-course experiments were performed in 5-d-old primary roots treated with 5 µM αNAA (Fig. 2B). Expression was measured relative to the housekeeping gene *myosin* (Genbank AC: 486090G09.x1) for each time point. The genes *rum1*, *lax1*, and *lax2* and all three *plt* genes (*plt1*, *bbm1*, and *hscf1*) were induced within 3h of αNAA exposure. Remarkably, expression of *arf8* and *arf37* was induced within 2h of αNAA exposure but returned to control levels 3h after auxin treatment. The transcription of *nac1* was not affected by αNAA exposure.

### RUM1 regulates vascular development

**Fig. 3.**
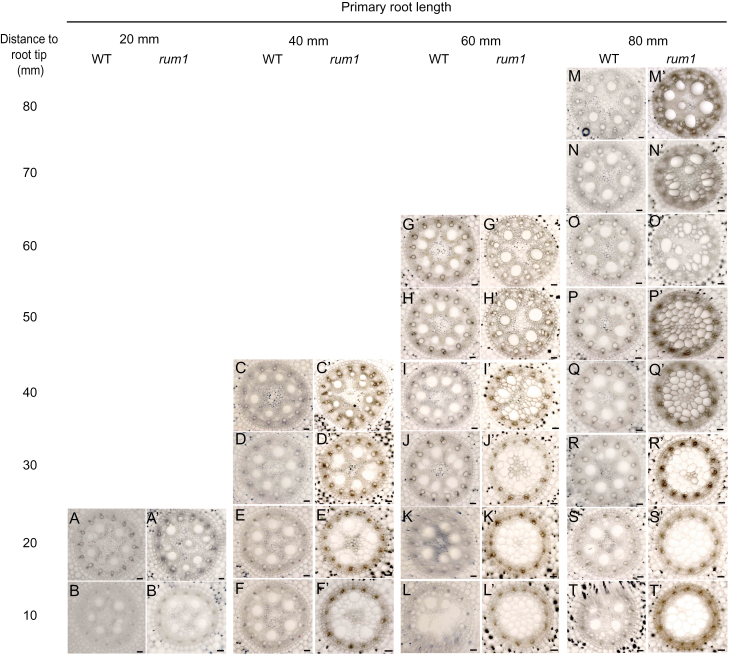
Series of transverse sections displaying the central cylinder of primary roots at different developmental stages: 20mm wild-type (A, B) and *rum1* (A′, B′) primary roots, 40mm wild-type (C–F) and *rum1* (C′–F′) primary roots, 60mm wild-type (G–L) and *rum1* (G′–L′) primary roots, 80mm wild-type (M–T) and *rum1* (M′–T′) primary roots. Sections were taken every 10mm and represent a single, representative primary root per genotype and developmental stage. The distances are indicated with reference to the root tip. Scale bar: 50 µm. (This figure is available in colour at *JXB* online.)

The homoeologues *arf8* (GRMZM2G034840) and *arf37* (GRMZM2G086949), which were significantly down-regulated in *rum1* (Fig. 2A) encode putative transcriptional activators. Their closest homologue in Arabidopsis is *MP*/*ARF5,* which regulates vascular development (Przemeck *et al.*, 1996; Hardtke and Berleth, 1998). To determine whether *rum1* mutants are impaired in vascular development, a comparative histological analysis of wild-type and *rum1* primary roots was performed at different developmental stages. In both genotypes, roots of 20, 40, 60, and 80mm length were dissected by serial transverse hand sections in 10mm increments (Fig. 3). In wild-type primary roots, at all four developmental stages (20, 40, 60, and 80mm), protoxylem elements, early metaxylem elements, and parenchymous pith cells surrounding the late metaxylem elements were well developed and properly arranged throughout development (Fig. 3A–T). By contrast, in primary roots of the mutant *rum1*, xylem elements and the pith cells were largely in disarray. Only the oldest part of the mutant roots at all four developmental stages displayed properly arranged xylem elements (20 mm: Fig. 3A′; 0 mm: Fig. 3C′; 60 mm: Fig. 3G′; 80 mm: Fig. 3M′). Moreover, in 60mm and 80mm *rum1* roots, only the oldest parts of the primary root (60 mm: Fig. 3G′–J′; 80 mm: Fig. 3M′–O′) displayed well differentiated xylem elements, while the younger parts of these roots (60 mm: Fig. 3K′–L′; 80 mm: Fig 3P′–T′) did not show any differentiated xylem elements. Hence, the magnitude of alterations in the vasculature of *rum1* correlated with the developmental stage at which these parts of the root were formed. In all analysed mutant roots, vascular differentiation was not affected in cells that were released from the meristematic zone soon after germination (Fig. 3A′, C′, G′, M′) whereas the defects became more severe the later these cells were released from the meristem.

The number of xylem elements and the number and size of pith cells was determined in representative cross-sections of 80mm *rum1* roots at a distance of 70mm from the root tip. In these sections, the number of early and late metaxylem elements was not altered, whereas the number of pith cells surrounding the late metaxylem was significantly reduced while their size in a radial direction was significantly enlarged compared with cells in the corresponding region of wild-type primary roots (Table 1).

**Table 1.** Quantification of xylem cell number and radial size in 80mm WT versus *rum1* roots at a distance of 70mm from the root tip

	Number of metaxylem cells		Pith cells around late metaxylem	
	Early	Late	Number	Size (µm)
WT	13±1	6±1	110±7	9.4±2.6
*rum1*	13±2	6±1	76±18^*a*^	20.6±12.5^*b*^

^*a*^ Number of WT vs rum1 pith cells: *p* ≤0.05.

^*b*^ Size of WT vs rum1 pith cells: *p* ≤0.01.

### Auxin control of vascular differentiation

**Fig. 4.**
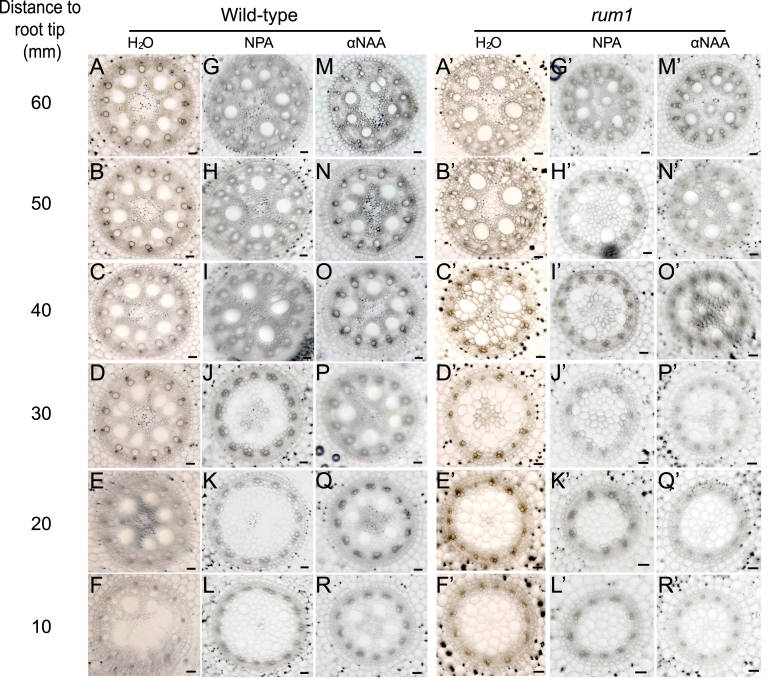
Effect of NPA and αNAA application on the formation of the vascular system of 60mm wild-type and *rum1* primary roots. Series of transverse sections displaying the central cylinder of wild-type (A–R) and *rum1* (A′–R′) primary roots grown in water (wild type: A–F; *rum1*: A′–F′), 10 µM NPA (wild type: G–L; *rum1*: G′–L′) and 0.1 µM αNAA (wild type: M–R; *rum1*: M′–R′). Sections were taken every 10mm and show a single, representative primary root per genotype and growth condition. The distances are indicated with reference to the root tip. Scale bar: 50 µm. (This figure is available in colour at *JXB* online.)

In a previous study, it was demonstrated that polar auxin transport was decreased by 83% in 3-d-old *rum1* primary roots (Woll *et al.*, 2005). To test the effect of auxin on vascular development in maize primary roots, wild-type and *rum1* seedlings were germinated either in the presence of the auxin transport inhibitor NPA (10 µM), the synthetic auxin αNAA (0.1 µM) or water. Primary roots of 60mm length were sectioned in 10mm increments (Fig. 4). In the mutant *rum1*, disorganized vascular patterns and enlarged pith cells were observed irrespective of water, NPA or αNAA treatment (Fig. 4A′–R′). Hence, treatment with αNAA, which can passively diffuse into the cells, did not recover the wild-type vascular phenotype in *rum1* primary roots (Fig. 4M′–R′). By contrast, while αNAA treatment of wild-type primary roots did not alter the vascular system (Fig. 4M–R), NPA treatment led to a similar disorganization in vascular patterning and enlarged pith cells surrounding the late metaxylem (Fig. 4J–L) as observed in the mutant *rum1*. This suggests that these defects are a consequence of reduced polar auxin transport.

### The *rum1* mutant displays enhanced lignification of aberrant pith cells

Mapman analyses of genes differentially expressed between wild-type and *rum1* primary roots revealed that three genes which encode key enzymes of lignin biosynthesis (4-COUMARATE-CoA LIGASE, 4CL; GRMZM2G16 5844, FERULATE 5-HYDROXYLASE, F5H; GRMZM2G 100158 and CINNAMYL ALCOHOL DEHYDROGENASE, CAD; GRMZM2G443445) were strongly induced in 2cm *rum1* primary roots (see Supplementary Table S3 at *JXB* online).

**Fig. 5.**
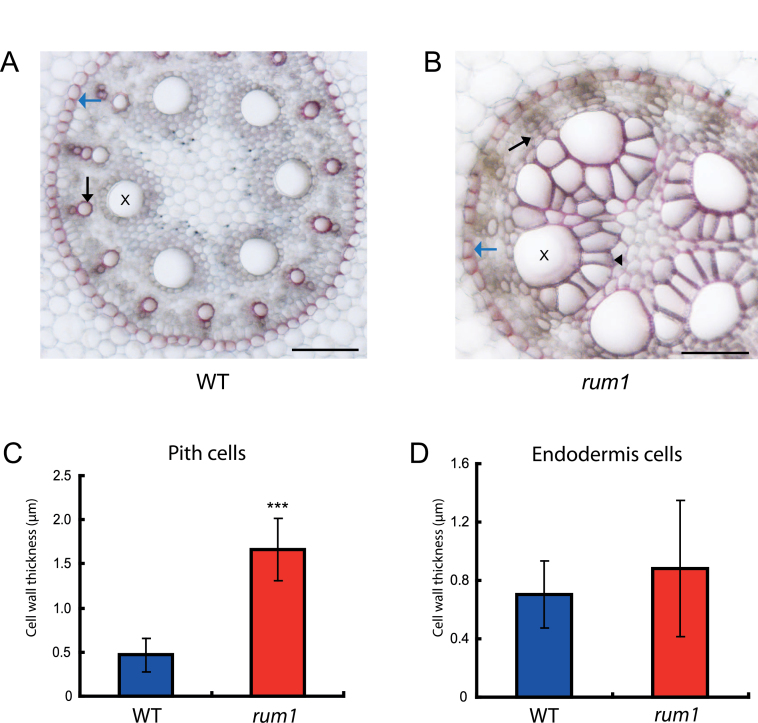
Analyses of lignification in wild-type and *rum1* primary roots. Cross-sections of the central region of 10-d-old wild-type (A) and *rum1* (B) primary roots. Phloroglucinol–HCl-stained lignified cell walls were stained in red. Lignin was detected in protoxylem and early metaxylem elements (black arrow), in enlarged pith cells (black arrowhead) surrounding the late metaxylem elements (x) in *rum1* and in endodermis cells forming Casparian strips (blue arrow). (C) Pith cells surrounding late metaxylem elements in the mutant *rum1* display significantly thicker cell walls than their wild-type counterparts (*n*=30, ***p ≤0.001). (D) Cell wall thickness of endodermis cells (*n*=18) is not significantly different between wild-type and *rum1* primary roots. (A,B) scale bar: 100 µm. (This figure is available in colour at *JXB* online.)

Secondary cell walls accumulate lignin or other secondary metabolites. Lignin deposition in the vasculature of wild-type (Fig. 5A) and *rum1* (Fig. 5B) primary roots was surveyed by phloroglucinol–HCl staining and identified by red staining of protoxylem and early metaxylem, and endodermis cells forming the Casparian strip. In contrast to wild-type primary roots (Fig. 5A), strong staining was detected in the larger and thickened pith cells surrounding the late metaxylem in *rum1* (Fig. 5B). Pith cell walls were significantly thicker in *rum1* than in wild-type primary roots (Fig. 5C), while cell wall thickness was not affected in *rum1* endodermis cells (Fig. 5D), which also deposit lignin. Hence, enhanced lignin deposition in mutant primary roots is probably not directly controlled by *rum1* but rather is a result of the defects during the differentiation of vascular cells.

## Discussion

In the present RNA-Seq study, 22 833 genes were expressed in the differentiation zone of 2cm primary roots of maize. A similar number of genes was expressed in a transcriptome analysis of maize primary roots by SAGE where it was extrapolated from 14 850 expressed genes that ~22 000 genes are active in the root tips of the cultivar FR697 (Poroyko *et al.*, 2005). These results suggest that at least ~55% of all high confidence gene models (ZmB73_RefGen_v2) are expressed in the differentiation zone and in root tips of young maize primary roots.

### RUM1 controls expression of genes associated with auxin signal transduction

Aux/IAA proteins such as RUM1 (von Behrens *et al.*, 2011) are auxin signal transduction regulators that control diverse aspects of plant development. In the present RNA-Seq study, *rum1* expression was significantly reduced in the differentiation zone of *rum1* primary roots compared with the wild type, confirming previous qRT-PCR experiments (von Behrens *et al.*, 2011). Several genes known to act downstream of *Aux/IAA* genes displayed RUM1-dependent expression and were auxin-inducible. A putative role of these genes in maize root development is suggested by the function of their orthologues in root formation in other plant species.

First, *nac1* (GRMZM2G081930), which encodes the NAC domain-containing protein 21/22 is down-regulated in the differentiation zone of *rum1* primary roots. The closest *Arabidopsis* homologue of this gene encodes the transcription factor NAC1 which controls lateral root initiation via an Aux/IAA-ARF-dependent auxin signalling module (Xie *et al*., 2000, 2002).

Three maize *plethora* (*plt*) genes were down-regulated in *rum1* including *plt1*, (GRMZM2G141638), *bbm1* (GRMZM2G366434), and *hscf1* (GRMZM2G139082). These genes encode members of the AP2/EREBP (APETALA2/ETHYLENE-RESPONSIVE ELEMENT BINDING PROTEIN) class transcription factor family. In *Arabidopsis*, several *PLT* genes are down-regulated in the mutant *slr/iaa14* (Aida *et al.*, 2004) which is blocked in lateral root formation. The maize *plt1* and *bbm1* genes are orthologues of *AtBBM,* which controls adventitious root formation (Srinivasan *et al.*, 2007). Moreover, maize *hscf1* is the closest relative of *Arabidopsis PLT3* and *PLT7* (see Supplementary Fig. S1 at *JXB* online), which are involved in the regulation of the auxin efflux transporter PIN1 (Prasad *et al.*, 2011). Although overall *pin1* levels were not altered in the *rum1* mutant (Woll *et al.*, 2005) cell-type specific alterations of *pin1* expression might explain the reduction in polar auxin transport by 83% in the *rum1* primary root.

Furthermore, in the present study the maize *like-aux1* (*lax1*, GRMZM2G129413) and *lax2* (GRMZM2G149481) which encode auxin influx carriers, were down-regulated in *rum1*. In *Arabidopsis*, the *AUX*/*LAX* gene family members *AUX1* and *LAX3* act concomitantly in lateral root initiation (Marchant *et al.*, 2002) and emergence (Swarup *et al.*, 2008).

Finally, maize *lbd24* (GRMZM2G075499) was down-regulated more than 4-fold in *rum1* primary roots. The closest homologue of maize *lbd24* is *Arabidopsis LBD16* (Majer and Hochholdinger, 2011). AtLBD16 controls asymmetric division of lateral root founder cells in *Arabidopsis* (Goh *et al.*, 2012), which is directly regulated by the SLR/IAA14-ARF7/ARF19-dependent auxin signalling module which controls *Arabidopsis* lateral root initiation (Okushima *et al.*, 2007).

In summary, in the present study the expression of several genes involved in auxin signal transduction was shown to be RUM1-dependent. In other species, orthologues of several of these genes are known to function in root development. Hence, genetic dissection of these candidate genes may provide a better understanding of the molecular processes involved in maize root formation.

### RUM1 controls auxin dependent xylem development

The *Arabidopsis* MP/ARF5 protein, which controls vascular development (Przemeck *et al.*, 1996; Hardtke and Berleth, 1998) is orthologous to the paralogous maize auxin signal transduction regulators ARF8 and ARF37 (von Behrens *et al.*, 2011; Wang *et al.*, 2012). In the present study, transcription of both *arf8* and *arf37* was almost completely repressed in the mutant *rum1*. These findings support the notion that transcription of *arf* genes is controlled by Aux/IAA proteins (Lau *et al.*, 2011). To survey if *arf8* and *arf37* repression affects xylem organization in *rum1* primary roots, a comparative histological analysis of *rum1* versus wild-type vasculature was undertaken. These experiments led to the observation that the fate of the vascular system in *rum1* primary roots depended on its developmental status.

The vascular system of the mutant *rum1* formed soon after germination at a distance of 10mm from the root apex displayed only minor defects in xylem organization (Fig. 3A′, C′, G′, M′). It could be hypothesized that, at this early stage of primary root development, the lack of *rum1* expression is compensated by another member of the maize *Aux/IAA* gene family. This notion is supported by a recent expression analysis of the maize *Aux/IAA* gene family reporting very low gene expression levels of *rum1* but much higher levels of other *Aux/IAA* genes such as *IAA14* in the meristematic zone of maize primary roots (Ludwig *et al.*, 2013). Moreover, at this developmental stage, auxin synthesized in the root apex (Feldmann, 1980) could compensate for the significantly reduced auxin amounts transported from the shoot to the root (Woll *et al.*, 2005). Subsequently formed xylem elements at a distance of 20–40mm from the root apex were in severe disarray and their surrounding pith parenchyma cells were significantly enlarged (Fig. 3B′, D′–F′, H′–J′; N′–O′). This observation suggests that RUM1 controls the development of distinct cells-types such as xylem elements and pith cell parenchyma. In *Arabidopsis*, it was reported that a mutually inhibitory feedback loop between auxin and cytokinin sets up the boundary between xylem and neighbouring procambial parenchyma cells (Bishopp *et al.*, 2011). Significant alterations in auxin and cytokinin signalling can affect the positions of these domains. Since the enlarged pith cells around the metaxylem elements are lignified, such as xylem cells (Fig. 5B), one could assume that these pith cells have altered their cell identity. However, in *Arabidopsis*, protoxylem cells are defined by high auxin signalling while the surrounding procambial cells are characterized by low auxin and high cytokinin signalling (Bishopp *et al.*, 2011). For instance, *Arabidopsis* mutants with significantly impaired auxin signalling such as *axr3* do not form xylem cells at all (Leyser *et al.*, 1996). Therefore, if one assumes a similar mechanism in maize, one would rather expect a reduction of the xylem elements in a mutant with compromised auxin signalling such as in *rum1*. Remarkably, the phenotype of disorganized xylem elements and enlarged lignified surrounding cells is only transiently observed during a defined developmental period. In continuously proliferating older primary roots of 60 or 80mm in length, no xylem elements were formed later in development (60 mm: Fig. 3K′–L′; 80 mm: Fig 3P′–T′). Hence, at this developmental stage, *rum1* roots display the same low auxin signalling phenotype as observed in the *Arabidopsis axr3* mutant (Leyser *et al.*, 1996).

It was demonstrated previously that the *Aux/IAA* mutant *rum1* displayed significantly reduced polar auxin transport (Woll *et al.*, 2005). A link between reduced polar auxin transport in *rum1* roots and the observed defects in vasculature formation was demonstrated by a treatment of wild-type roots with the polar auxin transport inhibitor NPA. NPA-treated wild-type roots showed the similar disarray in vascular organization as observed in the mutant *rum1*. This supports the notion that polar auxin transport is important for the differentiation of xylem elements and the surrounding pith cells in maize (Jansen *et al.*, 2012). By contrast, αNAA was not able to restore the wild-type phenotype in *rum1* primary roots. This is in line with the earlier observation that auxin treatment cannot induce lateral roots in the mutant *rum1* (Woll *et al.*, 2005), and supports the molecular model explaining RUM1 function (von Behrens *et al.*, 2011). According to this model, the rum1 mutant protein constitutively represses downstream auxin responsive genes even at high auxin levels (von Behrens *et al.*, 2011). The same effect and phenotype can be obtained in wild-type plants treated with NPA which inhibits polar auxin transport and thereby reduces cellular auxin levels.

A similar defect in vascular development has been demonstrated in tomato *IAA15* (Deng *et al.*, 2012). However, while *rum1* specifically controls vascular development in roots but not in the shoot, tomato *IAA15* regulates xylem development in the stem (Deng *et al.*, 2012).

### Excessive lignification of pith cell-walls in the primary root of the mutant *rum1*

The hydrophobic cell wall polymer lignin is deposited in endodermis and pith cells surrounding xylem elements to make them impermeable to water and to provide structural support (Vermerris *et al.*, 2010). Histochemical staining revealed excessive lignin deposition in enlarged pith cells of the mutant *rum1* while lignin deposition in endodermis cells was not significantly affected. Comparative RNA-Seq experiments of young maize primary roots revealed strong up-regulation of three lignin biosynthesis genes in mutant *rum1* roots. Recent studies revealed complex transcriptional networks that control lignin biosynthesis in addition to a plethora of developmental and environmental cues including various types of stress and auxin (Zhao and Dixon, 2011). The maize mutant *lrt1* (*lateralrootless1*) displays a similar phenotype as *rum1* (Hochholdinger and Feix, 1998). Both mutants fail to initiate lateral roots from the primary root. The *lrt1* gene has not yet been cloned but it has been demonstrated that it is not allelic with *rum1* (Woll *et al.*, 2005). A comparative proteome analysis of 9-d-old wild-type and *lrt1* primary roots revealed four proteins involved in lignin biosynthesis (including Caffeoyl-CoA-3-*O*-methyltransferase, CCoA-OMT) that were up-regulated in *lrt1* primary roots compared with wild-type primary roots (Hochholdinger *et al.*, 2004b). Similarly, a comparative transcriptome analysis of wild-type versus *rum1* pericycle cells revealed preferential expression of CCoA-OMT in mutant pericycle cells (Woll *et al.*, 2005).

Moreover, it has been demonstrated that inhibition of auxin transport in *Arabidopsis* resulted in dramatically increased numbers of cells with thickened secondary cell walls in stems which suggests that cell wall thickness is also regulated by auxin (Mattsson *et al.*, 1999). Therefore, drastically decreased polar auxin transport in *rum1* might lead to the mis-regulation of lignin biosynthesis genes resulting in thickened secondary cell walls in enlarged pith cells.

In summary, the data presented here indicate that the transcriptional repressor RUM1, in addition to its previously demonstrated function in regulating lateral and seminal root initiation, also controls vascular and pith cell differentiation and lignin deposition in these cells.

## Supplementary data

Supplementary data can be found at *JXB* online.

Supplementary Fig. S1. Phylogenetic tree of nine maize and eight *Arabidopsis plt* genes generated by ClustalV.

Supplementary Table S1. Summary of the RNA-Seq data of *rum1* and the wild type, and alignments to the B73 reference genome (ZmB73_RefGen_v2).

Supplementary Table S2. List of the 22 833 expressed genes and their characteristics.

Supplementary Table S3. Differentially expressed genes (Fc ≥2; FDR ≤1%) were functionally annotated using the MapMan software. Genes that were assigned to more than one functional class or subgroup are labelled in red. Fold changes (*rum1*/WT) are given as logarithmic (log_2_) values.

Supplementary Table S4. Determination of overrepresented and underrepresented functional classes among differentially expressed genes.

Supplementary Table S5. Sequences of oligonucleotide primers used for qRT-PCR analyses.

Supplementary Data
