# The *Aux/IAA* gene *rum1* involved in seminal and lateral root formation controls vascular patterning in maize (*Zea mays* L.) primary roots

**DOI:** 10.1093/jxb/erv354

**Published:** 2015-07-23

**Authors:** Yanxiang Zhang, Anja Paschold, Caroline Marcon, Sanzhen Liu, Huanhuan Tai, Josefine Nestler, Cheng-Ting Yeh, Nina Opitz, Christa Lanz, Patrick S. Schnable, Frank Hochholdinger

*Journal of Experimental Botany* Vol. 65, No. 17, pp. 4919–4930, 2014; doi:10.1093/jxb/eru249

A wild-type cross-section representing a distance of 20mm instead of 10mm from the root tip was displayed in Figure 3F. This error has been fixed in the corrected Figure 3 below.

**Fig. 3.**
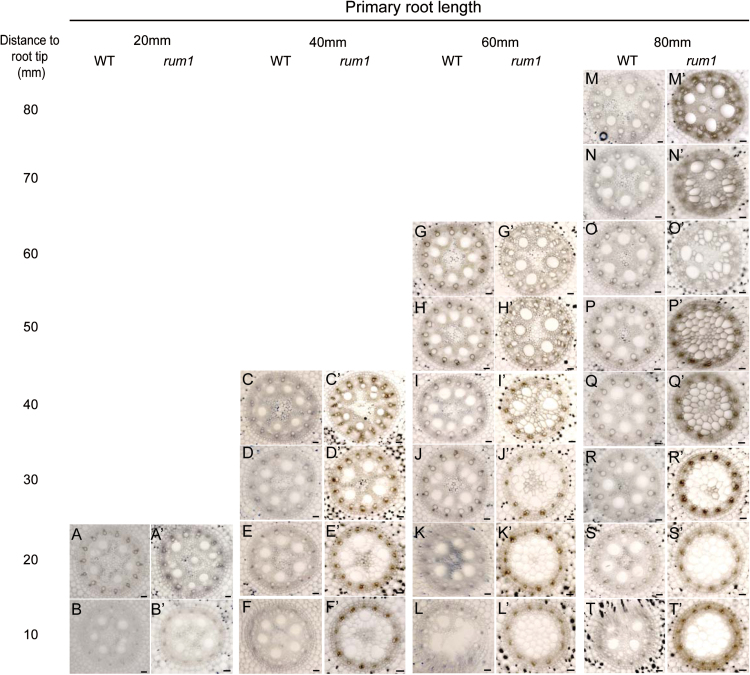
Series of transverse sections displaying the central cylinder of primary roots at different developmental stages: 20mm wild-type (A, B) and *rum1* (A′, B′) primary roots, 40mm wild-type (C–F) and *rum1* (C′–F′) primary roots, 60mm wild-type (G–L) and *rum1* (G′–L′) primary roots, 80mm wildtype (M–T) and *rum1* (M′–T′) primary roots. Sections were taken every 10mm and represent a single, representative primary root per genotype and developmental stage. The distances are indicated with reference to the root tip. Scale bar: 50 *μ*m. (This figure is available in colour at *JXB* online.)

