# Supplementary material for: The Aux/IAA gene rum1 involved in seminal and lateral root formation controls vascular patterning in maize (Zea mays L.) primary roots
Source: J Exp Bot. 2014 Jun 13;65(17):4919–30. doi: 10.1093/jxb/eru249 (PMC4144770; doi:10.1093/jxb/eru249)
Supplement: Supplementary Data [file supp_65_17_4919__index.html]

The Aux/IAA gene rum1 involved in seminal and lateral root formation controls vascular patterning in maize (Zea mays L.) primary roots — The Aux/IAA gene rum1 involved in seminal and lateral root formation controls vascular patterning in maize (Zea mays L.) primary roots — Supplementary Data 

# The *Aux/IAA* gene *rum1* involved in seminal and lateral root formation controls vascular patterning in maize (*Zea mays* L.) primary roots

## Supplementary Data

Data files

**Files in this Data Supplement:**

- Supplementary Data - Supplementary Data
- Supplementary Data - Supplementary Data
- Supplementary Data - Supplementary Data
