# Supplementary material for: The Aux/IAA gene rum1 involved in seminal and lateral root formation controls vascular patterning in maize (Zea mays L.) primary roots
Source: J Exp Bot. 2014 Jun 13;65(17):4919–30. doi: 10.1093/jxb/eru249 (PMC4144770; doi:10.1093/jxb/eru249)
Supplement: Supplementary Data [file supp_eru249_jexbot124693_file001.pdf]

The *Aux/IAA* gene *rum1* involved in seminal and lateral root formation controls vascular patterning in maize (*Zea mays* L.) primary roots

Yanxiang Zhang, Anja Paschold, Caroline Marcon, Sanzhen Liu, Huanhuan Tai, Josefine Nestler, Cheng-Ting Yeh, Nina Opitz, Christa Lanz, Patrick S. Schnable, and Frank Hochholdinger

Supplementary Figure S1. Phylogenetic tree of nine maize and eight Arabidopsis *plt* genes generated by ClustalV.

Supplementary Table S1. Summary of the RNA-Seq data of *rum1* and wild-type, and alignments to the B73 reference genome (ZmB73_RefGen_v2).

Supplementary Table S2. List of the 22,833 expressed genes and their characteristics.

Supplementary Table S3. Differentially expressed genes (FC ≥2; FDR ≤1%) were functionally annotated using the MapMan software. Genes that were assigned to more than one functional class or subgroup are labeled in red. Fold changes (*rum1*/WT) are given as logarithmic ($\log_2$) values.

Supplementary Table S4. Determination of overrepresented and underrepresented functional classes among differentially expressed genes.

Supplementary Table S5. Sequences of oligonucleotide primers used for qRT-PCR analyses.

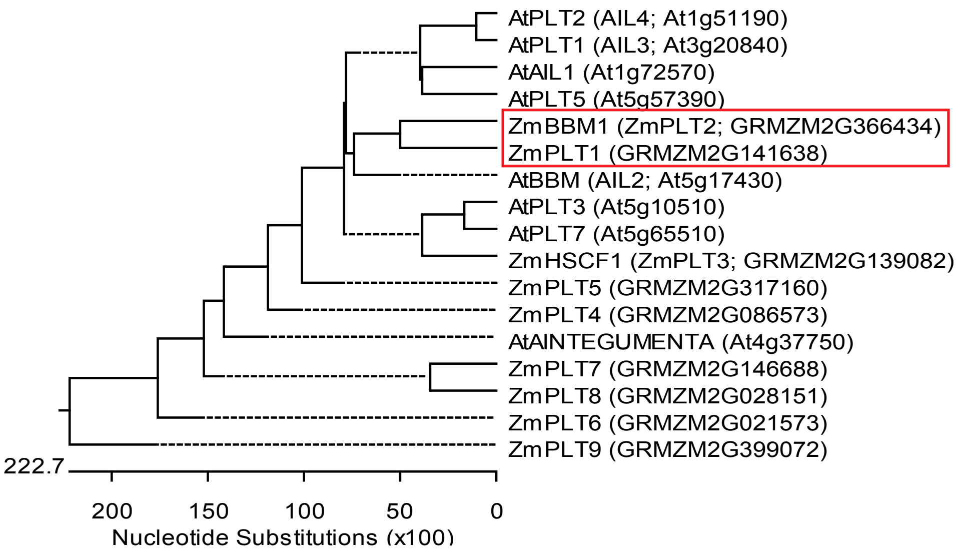

Supplemental Figure S1. Phylogenetic tree of nine maize and eight Arabidopsis plt genes generated by ClustalV.

Supplemental Table S1 Summary of the RNA-seq data of *rum1* and wild-type, and alignments to B73 reference genome (ZmB73_RefGen_v2).

Genotype	Sample Name	No. Raw Reads	Ave. No. Raw Reads	No. Trimmed Reads	Ave. No. Trimmed Reads	No. Reads Aligned	% Reads Aligned	Ave. % Reads Aligned	Reads in Gene Space	% Reads in Gene Space
WT	WT_1	15,044,869	15,086,826	9,466,791	9,630,318	6,852,187	72.4	73.1	6,453,938	94.2
	WT_2	11,337,249		7,282,077		5,220,452	71.7		4,922,088	94.3
	WT_3	23,338,101		15,054,876		10,921,434	72.5		10,286,556	94.2
	WT_4	10,627,083		6,717,529		5,087,182	75.7		4,799,450	94.3
rum1	*rum1*_1	22,606,675	15,567,135	11,159,477	7,693,614	8,468,447	75.9	76.1	7,977,142	94.2
	*rum1*_2	16,237,055		8,118,157		6,187,571	76.2		5,817,999	94.0
	*rum1*_3	8,260,770		4,057,333		3,098,951	76.4		2,943,005	95.0
	*rum1*_4	15,164,040		7,439,487		5,646,902	75.9		5,307,189	94.0

Supplemental Table S4: Determination of overrepresented or underrepresented functional classes among differentially expressed genes.

Bin[a]	Functional group	Expressed genes[b]	%	Differentially expressed genes[c]	%	Representation[d]	*p*-value
1	**Photosynthesis**	164	0.7%	36	1.2%	+	<0.001
2	**Major CHO metabolism**	119	0.5%	20	0.7%		0.11
3	**Minor CHO metabolism**	140	0.6%	23	0.8%		0.11
4	**Glycolysis**	91	0.4%	6	0.2%		
5	**Fermentation**	19	0.1%		0.0%		
6	**Gluconeogenesis/ glyoxylate cycle**	14	0.1%	2	0.1%		
7	**OPP**	34	0.1%	2	0.1%		
8	**TCA / org. transformation**	78	0.3%	4	0.1%		
9	**Mitochondrial e-transport / ATP synthesis**	128	0.5%	8	0.3%		
10	**Cell wall**	367	1.5%	77	2.6%	+	<0.001
11	**Lipid metabolism**	396	1.6%	36	1.2%		0.07
12	**N-metabolism**	40	0.2%	4	0.1%		
13	**Amino acid metabolism**	310	1.2%	26	0.9%		0.04
14	**S-assimilation**	12	0.0%		0.0%		
15	**Metal handling**	73	0.3%	22	0.7%	+	<0.001
16	**Secondary metabolism**	327	1.3%	60	2.0%	+	<0.001
17	**Hormone metabolism**	429	1.7%	60	2.0%		0.22
18	**Co-factor and vitamine met.**	77	0.3%	6	0.2%		
19	**Tetrapyrrole synthesis**	52	0.2%	7	0.2%		
20	**Stress**	780	3.1%	99	3.3%		0.60
21	**Redox regulation**	200	0.8%	22	0.7%		0.64
22	**Polyamine metabolism**	15	0.1%	2	0.1%		
23	**Nucleotide metabolism**	173	0.7%	21	0.7%		0.98
24	**Biodegradation of Xenobiotics**	29	0.1%	5	0.2%		

Bin[a]	Functional group	Expressed genes[b]	%	Differentially expressed genes[c]	%	Representation[d]	*p*-value
25	**C1-metabolism**	27	0.1%	2	0.1%		
26	**Miscelleanous**	1206	4.9%	222	7.4%	+	<0.001
27	**RNA**	2996	12.1%	259	8.6%	-	<0.001
28	**DNA**	513	2.1%	141	4.7%	+	<0.001
29	**Protein**	3482	14.0%	292	9.7%	-	<0.001
30	**Signalling**	1238	5.0%	153	5.1%		0.76
31	**Cell**	865	3.5%	127	4.2%		0.02
33	**Development**	531	2.1%	63	2.1%		0.87
34	**transport**	1069	4.3%	120	4.0%		0.38
35	**Not assigned**	8866	35.7%	1078	35.9%		0.80
	**DEG MapMan Sum**	**24860**	**100.0%**	**3005**	100.0%		
	**DEG Sum**	**22832**		**2801**			

[a] Each BIN consists of items of similar biological function.

[b] All genes that were expressed in the present study in wild-type or *rum1* primary roots.

[c] Subset of all expressed genes that are differentially expressed between wild-type and *rum1* (Fc ≥2; FDR ≤1%).

[d] Over (+) or underrepresentation (-) of differentially expressed genes in functional groups relative to the distribution of all expressed genes.

Supplemental Table S5: Sequences of oligonucleotide primers used in this study.

qRT-PCR:

**Name**	**Sequence**
Fw-primer (*rum1*-fw)	5´ CCTGCATCCAAGGAAGACAT 3´
Rv-primer (*rum1*-rv)	5´ CTTGACATCACGAACCATCG 3´
Fw-primer (*arf8*-fw)	5´ GCGAGAATGGCGTGCATGTC 3´
Rv-primer (*arf8*-rv)	5´ ACAAGAAGAGCCGCTGCGTG 3´
Fw-primer (*arf37*-fw)	5´ CTTCGGAAGTACAGCAGATG 3´
Rv-primer (*arf37*-rv)	5´ ACACTGTGCAGGCTGTAGAG 3´
Fw-primer (*nac1*-fw)	5´ CGACCATGTGGCAGTACAGCTC 3´
Rv-primer (*nac1*-rv)	5´ CAGTTGGTGCCTTGCCTCCTCA 3´
Fw-primer (*lax1*-fw)	5´ CTCCGTCGCCGATGATCAAC 3´
Rv-primer (*lax1*-rv)	5´ GCTGCGTGCTGATGACCAAG 3´
Fw-primer (*lax2*-fw)	5´ CATCACGATGCCATTCAACG 3´
Rv-primer (*lax2*-rv)	5´ CACGCACTACTGGTGGTAAG 3´
Fw-primer (*plt1*-fw)	5´ GAGCAACCACTACATCGGCA 3´
Rv-primer (*plt1*-rv)	5´ TGCAGACATCCTTCCGCCAC 3´
Fw-primer (*bbm1*-fw)	5´ CTAACAACTGGAGCTCTCCT 3´
Rv-primer (*bbm1*-rv)	5´ AACCAGAACCTGAGCACGTC 3´
Fw-primer (*hscf1*-fw)	5´ TCTGGATCTGCTGGCTACGAGG 3´
Rv-primer (*hscf1*-rv)	5´ GTCAGCGCATGCACCTACTCCA 3´
Fw-primer (cad-fw)	5´ CGATCCGTATCTCTCGCTCCT 3´
Rv-primer (cad-rv)	5´ GACAGGGATCATGTGAATGAGC-3´
Fw-primer (f5h-fw)	5´ GGAATGAAGCCCTCGGAGAT-3´
Rv-primer (f5h-rv)	5´GACAGAGCTAGAAAGCCACCAA 3´
Fw-primer (*myosin*-fw:486090G09.x1-5´)	5´ CAAGGAGAGACTCTGTGAGCTTCA 3´
Rv-primer (*myosin*-rv:486090G09.x1-3´)	5´ AGAAGGCCGTACAGGATCTTACC 3´
